# Augmenting Semantic Lexicons Using Word Embeddings and Transfer Learning

**DOI:** 10.3389/frai.2021.783778

**Published:** 2022-01-24

**Authors:** Thayer Alshaabi, Colin M. Van Oort, Mikaela Irene Fudolig, Michael V. Arnold, Christopher M. Danforth, Peter Sheridan Dodds

**Affiliations:** ^1^Advanced Bioimaging Center, University of California, Berkeley, Berkeley, CA, United States; ^2^Vermont Complex Systems Center, University of Vermont, Burlington, VT, United States; ^3^The MITRE Corporation, McLean, VA, United States; ^4^Department of Mathematics & Statistics, University of Vermont, Burlington, VT, United States; ^5^Department of Computer Science, University of Vermont, Burlington, VT, United States

**Keywords:** sentiment analysis, semantic lexicons, transformers, BERT, FastText, word embedding, labMT

## Abstract

Sentiment-aware intelligent systems are essential to a wide array of applications. These systems are driven by language models which broadly fall into two paradigms: Lexicon-based and contextual. Although recent contextual models are increasingly dominant, we still see demand for lexicon-based models because of their interpretability and ease of use. For example, lexicon-based models allow researchers to readily determine which words and phrases contribute most to a change in measured sentiment. A challenge for any lexicon-based approach is that the lexicon needs to be routinely expanded with new words and expressions. Here, we propose two models for automatic lexicon expansion. Our first model establishes a baseline employing a simple and shallow neural network initialized with pre-trained word embeddings using a non-contextual approach. Our second model improves upon our baseline, featuring a deep Transformer-based network that brings to bear word definitions to estimate their lexical polarity. Our evaluation shows that both models are able to score new words with a similar accuracy to reviewers from Amazon Mechanical Turk, but at a fraction of the cost.

## 1. Introduction

In computational linguistics and natural language processing (NLP), sentiment analysis involves extracting emotion and opinion from text data. There is an increasing demand for sentiment-aware intelligent systems. The growth of sentiment-aware frameworks in online services can be seen across a vast, multidisciplinary set of applications (Nasukawa and Yi, 2003; Medhat et al., 2014; Bakshi et al., 2016).

With the modern volume of text data—which has long rendered human annotation infeasible—automated sentiment analysis is used, for example, by businesses in evaluating customer feedback to make informed decisions regarding product development and risk management (Turney, 2002; Cabral and Hortacsu, 2010). Combined with recommender systems, sentiment analysis has also been used with the intent to improve consumer experience through aggregated and curated feedback from other consumers, particularly in retail (Kumar and Lee, 2006; Tang et al., 2009; Yu et al., 2013), e-commerce (Bhatt et al., 2015; Haque et al., 2018), and entertainment (Terveen et al., 1997; Pang et al., 2002).

Beyond applications in industry, sentiment analysis has been widely applied in academic research, particularly in the social and political sciences (Chen et al., 2021). Public opinion, e.g., support for or opposition to policies, can be potentially gauged from online political discourse, giving policymakers an important window into public awareness and attitude (Laver et al., 2003; Thomas et al., 2006). Sentiment analysis tools have shown mixed results in forecasting elections (Tumasjan et al., 2010) and monitoring inflammatory discourse on social media, with vital relevance to national security (Pang and Lee, 2008). Sentiment analysis has also been used in the public health domain (Coppersmith et al., 2014; Yadollahi et al., 2017; Gohil et al., 2018), with recent studies analyzing social media discourse surrounding mental health (Bathina et al., 2021; Stupinski et al., 2021), disaster response and emergency management (Beigi et al., 2016).

The growing number of applications of sentiment-aware systems has led the NLP community in the past decade to develop end-to-end models to examine short- and medium-length text documents (Wilson et al., 2005; Feldman, 2013), particularly for social media (Pak and Paroubek, 2010; Agarwal et al., 2011; Korkontzelos et al., 2016). Some researchers have considered the many social and political implications of using AI for sentiment detection across media (Crawford, 2019; Crawford and Paglen, 2021). Recent studies highlight some of the implicit hazards of crowdsourcing text data (Shmueli et al., 2021), especially in light of the latest advances in NLP and emerging ethical concerns (Conway and O'Connor, 2016; Hovy and Spruit, 2016). Identifying potential racial and gender disparity in NLP models is essential to develop better models (Tatman, 2017).

Sentiment analysis tools fall into one of two groups, depending on their definition of sentiment and their model for its estimation. One of the more popular paradigms is discrete classification, where sentiment is divided into several classes (e.g., positive, negative) and pieces of text are associated with each class. However, sometimes a continuous measure is desired, requiring a spectrum of sentiment scores rather than sentiment classes (Thelwall et al., 2010). This more nuanced sentiment scoring paradigm has been widely adopted for e-commerce, movies, and restaurant reviews (Snyder and Barzilay, 2007).

Sentiment analysis models largely derive from two major paradigms: 1. Lexicon-based models and 2. Contextual models. Lexicon-based models compute sentiment scores based on sentiment dictionaries (sentiment lexicons) typically constructed by human annotators (Taboada et al., 2011; Dodds et al., 2015; Augustyniak et al., 2016). A sentiment lexicon contains not only terms that express a particular sentiment/emotion, but also terms that are associated with a particular sentiment/emotion (denotation vs. connotation). Contextual models, on the other hand, extrapolate semantics by converting words to vectors in an embedding space, and learning from large-scale annotated datasets to predict sentiment based on co-occurrence relationships between words (Wilson et al., 2005; Pak and Paroubek, 2010; Agarwal et al., 2011; Feldman, 2013; Socher et al., 2013b). Contextual models have the advantage in differentiating multiple meanings, as in the case of “The dog is *lying* on the beach” vs. “I never said that—you are *lying*,” while lexicon-based models usually have a single score for each word, regardless of usage. Despite the flexibility of contextual models, their results can be difficult to interpret, as the high-dimensional latent space in which they are embedded renders explanation difficult. The ease of use and transparent comprehension of lexicon-based models help explain their continued popularity (Pang and Lee, 2008; Taboada et al., 2011; Dodds et al., 2015). For example, while the linguistic mechanisms leading to change in sentiment may be hard to explain with word embeddings, one can straightforwardly use lexicon scores to reveal the words contributing to shifted sentiment (Dodds et al., 2011; Reagan et al., 2017; Gallagher et al., 2021).

A major challenge for the simpler and more interpretable lexicon-based models, however, is the time and financial investment associated with maintaining them. Sentiment lexicons must be updated regularly to mitigate the out-of-vocabulary (OOV) problem—words and phrases that were either not considered or did not exist when the dictionaries were originally constructed (Riloff, 1996). While researchers show general sentiment trends are observable unless the lexicon does not have enough words, having a versatile dictionary with specialized and rarely used words improves the signal (Dodds and Danforth, 2010; Reagan et al., 2017). Notably, language is an evolving sociotechnical phenomenon. New words and phrases are created constantly, especially on social media (Alshaabi et al., 2021a). Word usage changes over time. New words are created, old words lose popularity, and the meaning of words can change. For example, the word “covid” grew to be the most narratively trending *n*-gram in reference to the global Coronavirus outbreak during February and March 2020 (Alshaabi et al., 2021b).

Sentiment analysis applications are often developed to investigate bipolar relationships (e.g., positive–negative, happy–sad, excited–bored). These bipolar relationships are conveniently handled by binary classification systems, however, such a formalization leads to multiple varieties of neutral sentiment (Colhon et al., 2017). Many sentiment analysis applications avoid, ignore, or remove text with neutral sentiment. Excluding neutral sentiment text during training can have significant impacts on trained models, which are often confused by or uncertain of neutral sentiment text (Koppel and Schler, 2006). For classification-based applications, explicitly representing neutral sentiment as a third class can improve model performance (Ribeiro et al., 2016). Humans process emotionally charged words differently than neutral words, thus sentiment analysis model may find success via similar processes Kissler and Herbert (2013).

In this work, we propose an automated framework extending sentiment for semantic lexicons to OOV words, reducing the need for crowdsourcing scores from human annotators, a process that can be time-consuming and expensive. Although our framework can be used in a more general sense, we focus on predicting *happiness scores* based on the labMT dataset (Dodds et al., 2015). This dataset was constructed from human ratings of the “happiness” of words on a continuous scale, averaging scores from multiple annotators for more than 10,000 words. We discuss this dataset in detail in section 3.1. In section 2, we discuss recent developments using deep learning in NLP, and how they relate to our work. We introduce two models, demonstrating accuracy on par with human performance (see section 3 for technical details). We first introduce a baseline model—a neural network initialized with pre-trained word embeddings—to gauge happiness scores. Second, we present a deep Transformer-based model that uses word definitions to estimate their sentiment scores. We will refer to our models as the “Token” and “Dictionary” models, respectively. We present our results and model evaluation in section 4, highlighting how the models perform compared with reviewers from Amazon's Mechanical Turk. Finally, we highlight key limitations of our approach, and outline some potential future developments in concluding remarks.

## 2. Related Work

Word embeddings are abstract numerical representations of the relationships between words, derived from statistics on individual corpora, and encoding language patterns so that concepts with similar semantics have similar representations (Bengio et al., 2003). Researchers have shown that efficient representations of words can both express meanings and preserve context (Maas et al., 2011; Hollis and Westbury, 2016; Hollis et al., 2017; Li et al., 2017). While there are many ways to construct word embedding models (e.g., matrix factorization), we often use the term to refer to a specific class of word embeddings that are learnable via neural networks.

Word2Vec is one of the key breakthroughs in NLP, introducing an efficient way for learning word embeddings from a given text corpus (Mikolov et al., 2013a,b). At its core, it builds off of a simple idea borrowed from linguistics and formally known as the “distributional hypothesis”—words that are semantically similar are also used in similar ways, and likely to appear with similar context words (Harris, 1954).

Starting from a fixed vocabulary, we can learn a vector representation for each word via a shallow network with a single hidden layer trained in one of two fashions (Mikolov et al., 2013a,b). Both approaches formalize the task as a unsupervised prediction problem, whereby an embedding is learned jointly with a network that is trained to either predict an anchor word given the words around it (i.e., continuous bag-of-words (CBOW)), or by predicting context words for an anchor word (i.e., skip-gram) (Mikolov et al., 2013a). Both approaches, however, are limited to local context bounded by the size of the context window. Global Vectors (GloVe) addresses that problem by capturing corpus global statistics with a word co-occurrence probability matrix (Pennington et al., 2014).

While Word2Vec and GloVe offer substantial improvements over previous methods, they both fail to encode unfamiliar words—tokens that were not processed in the training corpora. FastText refines word embeddings by supplementing the learned embedding matrix with subwords to overcome the challenge of OOV tokens (Bojanowski et al., 2017; Joulin et al., 2017). This is achieved by training the network with character-level *n*-grams (*n* ∈ {3,4,5,6}), then taking the sum of all subwords to construct a vector representation for any given word. Although the idea behind FastText is rather simple, it presents an elegant solution to account for rare words, allowing the model to learn more general word representations.

A major shortcoming of the earlier models is their inability to capture contextual descriptions of words as they all produce a fixed vector representation for each word. In building context-aware models, researchers often use fundamental building blocks such as recurrent neural networks (RNN) (Rumelhart et al., 1986)—particularly long short-term memory (LSTM) (Hochreiter and Schmidhuber, 1997)—that are designed to process sequential data. Many methods have provided incremental improvements over time (Chen et al., 2017; Lee et al., 2017; Peters et al., 2017). ELMo is one of the key milestones toward efficient contextualized models, using deep bi-directional LSTM language representations (Peters et al., 2018).

In late 2017, the advent of deep attention-based models, dubbed transformers, rapidly changed the landscape in the NLP community (Vaswani et al., 2017). The encoder-decoder framework, powered by attention blocks, enables faster processing of the input sequence while also preserving context (Vaswani et al., 2017). Recent adaptations of the building blocks of Transformers continue to break records, improving the state-of-the-art across all NLP benchmarks with recent applications to computer vision and pattern recognition (Dosovitskiy et al., 2021).

Exploiting the versatile nature of Transformers, we observe the emergence of a new family of language models widely known as “self-supervised” including as bidirectional encoders (e.g., BERT) (Devlin et al., 2019), and left-to-right decoders (e.g., GPT) (Radford et al., 2018). Self-supervised language models are pre-trained by masking random tokens in the unlabeled input data and training the model to predict these tokens. Researchers leverage recent subword tokenization techniques, such as WordPiece (Wu et al., 2016), SentencePiece (Kudo and Richardson, 2018), and Byte Pair Encoding (BPE) (Sennrich et al., 2016), to overcome the challenge of rare and OOV words. Subtle contextualized representations of words can be learned by predicting whether sentence B follows sentence A (Devlin et al., 2019). Pre-trained language models can then be fine-tuned using labeled data for downstream NLP tasks, such as named entity recognition, question answering, text summarization, and sentiment analysis (Radford et al., 2018; Devlin et al., 2019).

Recent advances in NLP continue to improve the language facility of Transformer-based models. The introduction of XLNet (Yang et al., 2019) is another remarkable breakthrough that combines the bi-directionality of BERT (Devlin et al., 2019) and the autoregressive pre-training scheme from Transformer-XL (Dai et al., 2019). While the current trend of making ever-larger and deeper language models shows an impressive track record, it is arguably unfruitful to maintain unreasonably large models that only giant corporations can afford to use due to hardware limitations (Thompson et al., 2020). Vitally, less expensive language models need to be both computationally efficient and exhibit performance on par with larger models. Addressing that challenge, researchers proposed clever techniques of leveraging knowledge distillation (Hinton et al., 2015) to train smaller and faster models [e.g., DistilBERT (Sanh et al., 2019)]. Similarly, efficient parameterization strategies via sharing weights across layers can also reduce the size of the model while maintaining state-of-the-art results [e.g., ALBERT (Lan et al., 2020)].

Previous work on automatic sentiment lexicon generation (ASLG) has used a variety of heuristics to assign sentiment scores to OOV words. Most ASLG methods start with a seed lexicon containing words of known sentiment, then use a distance function to propagate sentiment scores from known words to unknown words. Word co-occurrence frequencies (Turney and Littman, 2003; Kiritchenko et al., 2014) and shortest path distances within a semantic word graph (Qiu et al., 2009; Baccianella et al., 2010; San Vicente et al., 2014) [such as WordNet (Fellbaum, 1998)] were common distance functions in earlier work. More recently, distance functions based on learned word embeddings have gained popularity (Tang et al., 2014; Wang et al., 2016; Ljubešić et al., 2018; Thavareesan and Mahesan, 2020). The outputs of word embedding models usually need to be projected into a lower dimension before they can be used for ASLG. This can be done using a variety of machine learning models, though linear models are likely one of the most popular options (Qiu et al., 2009; Amir et al., 2015; Wang et al., 2016; Li et al., 2017; Alshari et al., 2018; Ljubešić et al., 2018; Thavareesan and Mahesan, 2020). Amir et al. (2015) proposed the use of a support vector regressor (SVR) trained with CBOW (Mikolov et al., 2013a) or GloVe (Pennington et al., 2014) word embeddings, finding that the SVR model out performed various linear models [e.g., Lasso (Yuan and Lin, 2006), Ridge (Hoerl and Kennard, 1970), ElasticNet (Zou and Hastie, 2005) regressors] on the labMT lexicon. However, their models only predicted a binary sentiment polarity (ŷ ∈ [0, 1]), rather than continuous scores. Li et al. (2017) extended their work, proposing a class of linear regression models trained with word embeddings to predict affective meanings in several sentiment lexicons such as ANEW (Bradley and Lang, 1999), VAD (Mohammad, 2018). Darwich et al. (2019) present an excellent review of ASLG.

Many of the human engineered heuristics used in previous work on ASLG can be largely automated via clever application of new machine learning techniques. Sentiment analysis knowledge bases can be constructed using graph-mining and multi-dimensional scaling techniques (Bajpai et al., 2016). Once constructed, these knowledge bases allow for the application of a host of additional methods. Neural tensor networks can be used for knowledge base completion, inferring relationships that were missed during construction (Socher et al., 2013a). Graph neural networks can create rich features from the relationships captured in knowledge bases, allowing sentiment analysis models to handle complex context-based problems (Dowlagar and Mamidi, 2021; Liao et al., 2021; Yang et al., 2021). Ensembles of symbolic and sub-symbolic AI can be used to cover the individual weaknesses of each method (Cambria et al., 2020).

Building on the many of the models discussed above, we develop a framework for augmenting semantic lexicons using word embeddings and pre-trained large language models. Our models output continuous valued sentiment scores that can represent degrees of negative, neutral, and positive sentiment. Our tool reduces the need for crowdsourcing scores from human annotators while still providing similar, and often better, results compared with random reviewers from Amazon Mechanical Turk at a fraction of the cost.

## 3. Materials and Methods

We propose two models for predicting happiness scores for the labMT lexicon (Dodds et al., 2015)—a general-purpose sentiment lexicon used to measure happiness in text corpora (see section 3.1 for more details).

Our first model is a neural network initialized with pre-trained FastText word embeddings. The model uses fixed word representations to gauge the happiness score for a given expression, enabling us to augment the labMT dataset at a low cost. For simplicity, we will refer to this model as the Token model.

Bridging the link between lexicon-based and contextualized models, we also propose a deep Transformer-based model that uses word definitions to estimate their happiness scores—namely, the Dictionary model. The contextualized nature of the input data allows our model to accurately estimate the expressed happiness score for a given word based on its lexical meaning.

We implement our models using Tensorflow (Abadi et al., 2016) and Transformers (Wolf et al., 2020). See section 3.2 and section 3.3 for additional details of our Token and Dictionary models, respectively. Our source code, along with pre-trained models, are publicly available via our GitLab repository (https://gitlab.com/compstorylab/sentiment-analysis).

### 3.1. Data

In this study, we use the labMT dataset as an example sentiment lexicon to test and evaluate our models (Dodds et al., 2015). The labMT lexicon contains roughly ten thousand unique words—combining the five thousand most frequently used words from New York Times articles, Google Books, Twitter messages, and music lyrics (Dodds et al., 2015). It is a lexicon designed to gauge changes in the happiness (i.e., valence or hedonic tone) of text corpora. Happiness is defined on a continuous scale *h* ∈ {1 → 9}, where 1 bounds the most negative (sad) side of the spectrum, and 9 is the most positive (happy). Ratings for each word are crowdsourced via Amazon Mechanical Turk (AMT), taking the average score *h*_*avg*_ from 50 reviewers to set a happiness score for any given word. For example, the words “suicide,” “terrorist,” and “coronavirus” have the lowest happiness scores, while the words “laughter,” “happiness,” and “love” have the highest scores. Function and stop words along with numbers and names tend to have neutral scores (*h*_*avg*_ ≈ 5), such as “the,” “fourth,” “where,” and “per.”

The labMT dataset also powers the Hedonometer, an instrument quantifying daily happiness on Twitter (Dodds et al., 2011). Over the past few years, the labMT lexicon was updated to include new words that were not found in the original survey [e.g, terms related to the COVID19 pandemic (Alshaabi et al., 2021b)].

We are particularly interested in this dataset because it also provides the standard deviation of human ratings for each word, which we use to evaluate our models. In this work, we propose two models to estimate *h*_*avg*_ using word embeddings, and thus provide an automated tool to augment the labMT dataset both reliably and efficiently.

**Figure 1:**
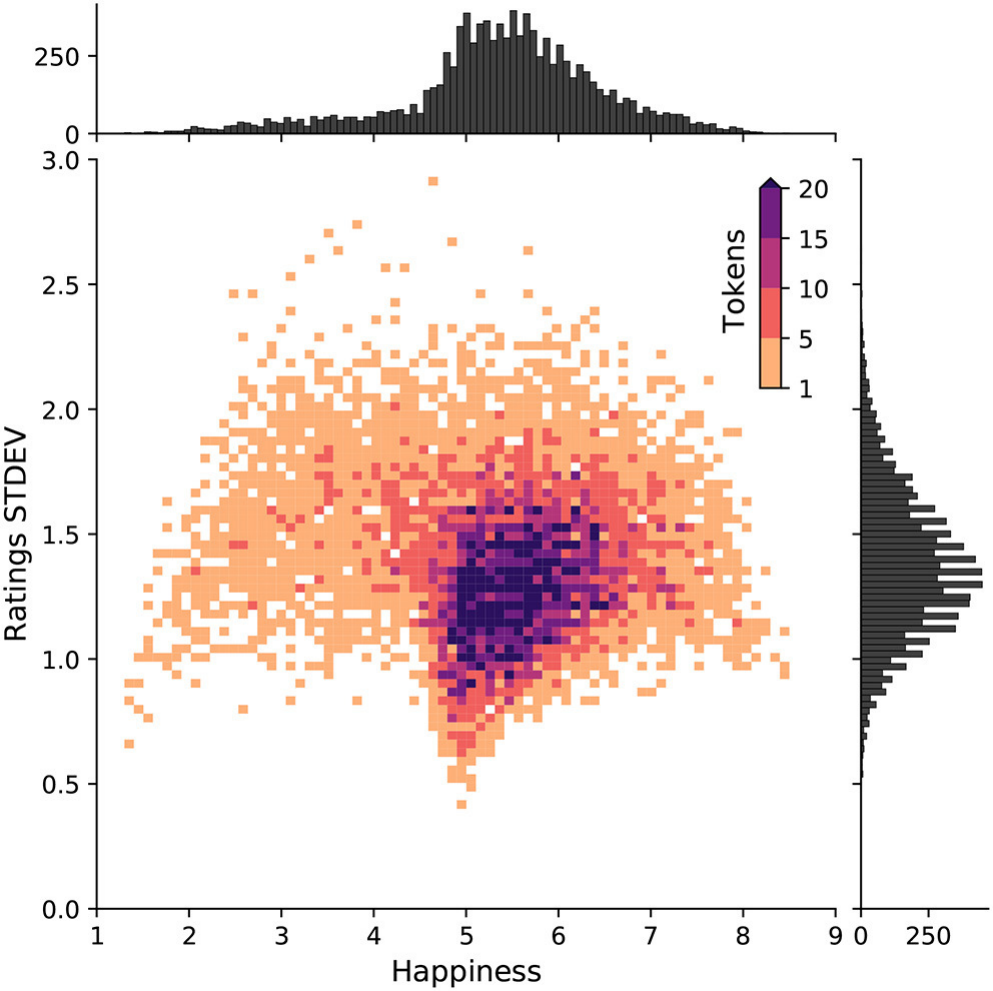
Emotional valence of words and uncertainty in human ratings of lexical polarity. A 2D histogram of happiness *h*_*avg*_ and standard deviation of human ratings for each word in the labMT dataset. Happiness is defined on a continuous scale from 1 to 9, where 1 is the least happy and 9 is the most. Words with a score between 4 and 6 are considered neutral. While the vast majority of words are neutral, there is a positive bias in human language (Dodds et al., 2015). The average standard deviation of human ratings for estimating the emotional valence of words in the labMT dataset is 1.38.

In Figure 1, we display a 2D histogram of the human rated happiness scores in the labMT dataset. The figure highlights the degree of uncertainty in human ratings of the emotional valence of words. For example, the word “the” has an average happiness score of *h*_*avg*_ = 4.98, with standard deviation of σ = 0.91, while the word “hahaha” has a happier score with *h*_*avg*_ = 7.94 and σ = 1.56. Some words also have a relatively large standard deviation such as “church” (*h*_*avg*_ = 5.48, σ = 1.85), and “cigarettes” (*h*_*avg*_ = 3.31, σ = 2.6).

While the majority of words are neutral, with a score between 4 and 6, we still observe a human positivity bias in the English language (Dodds et al., 2015; Aithal and Tan, 2021). On average, the standard deviation of human ratings is 1.38. In our evaluation (section 4), we show how our models perform relative to the uncertainty observed in human ratings.

### 3.2. Token Model

Our first model uses a neural network that learns to map words from the labMT lexicon to their corresponding sentiment scores. While still being able to learn a non-linear mapping between the words and their happiness scores, the model only considers the individual words as input—enriching its internal utility function with subword representations to estimate the happiness score.

**Figure 2:**
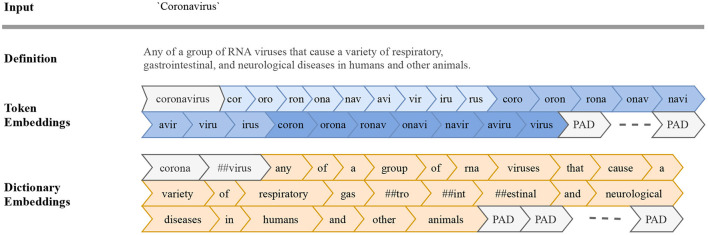
Input sequence embeddings. We use two encoding schemes to prepare input sequences for our models: token embeddings (blue) and dictionary embeddings (orange) for our Token and Dictionary models, respectively. Given an input word (e.g., “coronavirus,”) we first break the input token into character-level *n*-grams (*n* ∈ {3, 4, 5}). The resulting sequence of *n*-grams along with the original word at the beginning of the embeddings are used in our Token model. Sequences shorter than a specified length are appended with PAD, a padding token ensuring a universal input size. For our Dictionary model, we first look up a dictionary definition for the given input. We then process the input word along with its definition into subwords using WordPiece (Wu et al., 2016). Uncommon and novel words are broken into subwords, with double hashtags indicating that the given token is not a full word.

The input word is first processed into a token embedding—sequentially breaking each word into its equivalent character-level *n*-grams whereby *n* ∈ {3,4,5} (see Figure 2 for an illustration). English words have an average length of 5 characters (Miller et al., 1958; Mayzner and Tresselt, 1965), which would yield 6 unique character-level *n*-grams given our tokenization scheme. While we did try shorter and longer sequences, we fix the length of the input sequence to a size of 50 and pad shorter sequences to ensure a universal input size. We choose a longer sequence length to allow us to encode longer *n*-grams and rare words.

We then pass the token embeddings to a 300-dimensional embedding layer. We initialize the embedding layer with weights trained with subword information on Common Crawl and Wikipedia using FastText (Bojanowski et al., 2017). In particular, we use weights from a pre-trained model using CBOW with character-level *n*-grams of length 5 and a window size of 5 and 10 (https://fasttext.cc/docs/en/english-vectors.html).

The output of the embedding layer is pooled down and passed to a sequence of three dense layers of decreasing sizes: 128, 64, and 32, respectively. We use a rectified linear activation function (ReLU) for all dense layers. We also add a dropout layer after each dense layer, with a 50% dropout rate to add stochasticity to the model, allowing for a simple estimate of uncertainty using the standard deviation of the network's predictions (Srivastava et al., 2014).

**Figure 3:**
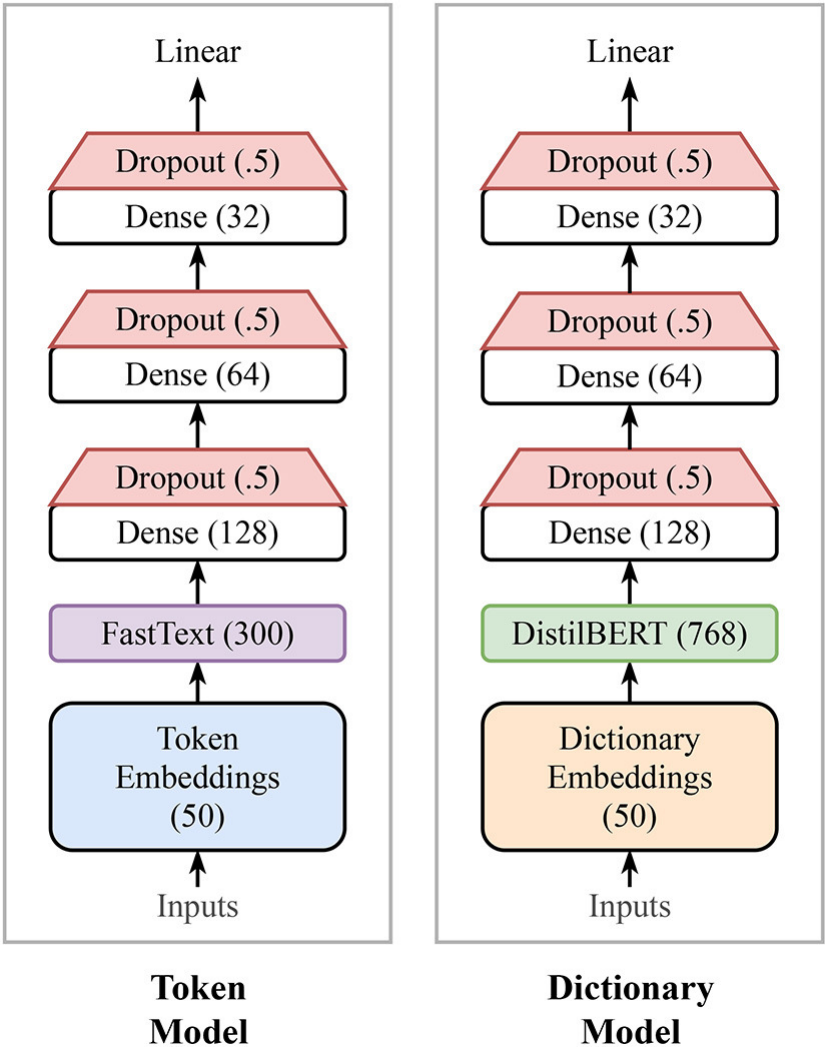
Model architectures. Our first model is a neural network initialized with pre-trained word embeddings to estimate happiness scores. Our second model, is a deep Transformer-based model that uses word definitions to estimate their sentiment scores. See section 3.2 and 3.3 for further technical details of each model, respectively. Note the Token model is considerably smaller with roughly 10 million trainable parameters compared with the Dictionary model that has a little over 66 million parameters.

We experimented with a few different layout configurations, finding that making the network either wider or deeper has minimal effect on the network performance. Therefore, we choose to keep our model rather simple with roughly 10 million trainable parameters. The output of the last dense layer is finally passed over to a single output layer with a linear activation function to regress a sentiment score between 1 and 9. See Figure 3 for a simple diagram of the model architecture.

### 3.3. Dictionary Model

Historically, lexicon-based models have only considered simple statistical methods to estimate the emotional valence of words. Here, we try to bridge the connection between the conventional techniques among the community and recent advances in NLP.

For our second model, we use a contextualized Transformer-based language model to estimate the sentiment score for a given word based on its dictionary definition. While still predicting scores for individual words, we now do so by augmenting each word with its expressed meaning(s) from a general dictionary. Given an input word, we look up its definition via a free online dictionary API available at https://dictionaryapi.dev.

The average length of definitions for the words found in labMT is roughly 38 words. We choose a maximum definition length of 50 words—which covers the 75th percentile of that distribution—to ensure that words with multiple definitions are adequately represented. While increasing the sequence length beyond 50 did not improve our accuracy, it increases the model complexity slowing our training and inference time substantially. Therefore, we fix the length of word definitions to a maximum of 50 words. We pad shorter sequences, and truncate words 51 and beyond to ensure a fixed input size.

We estimate the sentiment of each labMT word as follows. The word, along with its definition, is processed into dictionary embeddings by breaking each word into subwords based on their frequency of usage using WordPiece (Wu et al., 2016). This is a widely adopted tokenization technique that breaks uncommon and novel words into subwords, which reduces the vocabulary size of language models and enables them to handle OOV tokens. Other tokenization models will give similar results (Kudo and Richardson, 2018). We only use the word as input to our model for terms without definitions.

In principle, the dictionary embeddings can be passed to a vanilla Transformer model [e.g., BERT (Devlin et al., 2019), XLNet (Yang et al., 2019)]. However, we prefer more manageable models (i.e., smaller and faster) due to their efficiency while maintaining state-of-the-art results. We tried both ALBERT (Lan et al., 2020) and DistilBERT (Sanh et al., 2019). Both models have equivalent performance on our task. The output of the model's pooling layer is passed to a sequence of three dense layers of decreasing sizes with dropout applied after each layer—similar to our approach in the Token model. Finally, the output of the last dense layer is projected down to a single output value that servers as the sentiment score prediction.

The Token model is considerably lighter in terms of memory usage, and faster in terms of training and inference time than the Dictionary model. Our current configuration of the Token model results in roughly 10 million trainable parameters compared with the Dictionary model that has over 66 million parameters.

## 4. Results

### 4.1. Ensemble Learning and *k*-Fold Cross-Validation

In the deep learning community, particularly in the NLP domain, it is common to scale up the number of parameters in successful models to eke out additional performance gains. The effectiveness of this approach tends to be correlated with the amount of training data available (i.e., larger models are more effective when trained on larger data sets). With the limited size of our training set, we needed alternative techniques to increase the performance of our models. Ensemble learning is a widely known and adopted family of methods in which the average performance of an ensemble is shown to be both less biased and better than the individual models (Hansen and Salamon, 1990; Krogh and Vedelsby, 1994).

**Figure 4:**
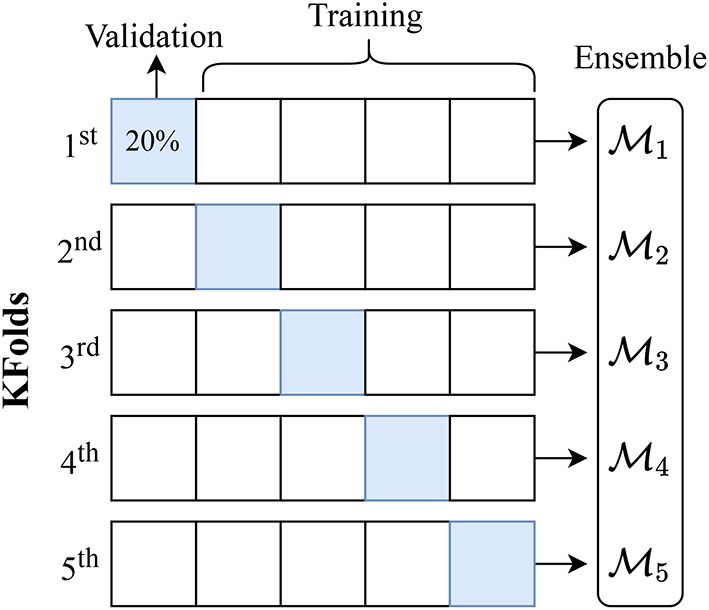
Ensemble learning and *k*-fold cross-validation. Using an 80/20 split for training/validation, we train our models for a maximum of 500 epochs per fold for a total of 5 folds. We use the model trained from each fold to build an ensemble because the average performance of an ensemble is less biased and better than the individual models.

First, we randomly subsample our dataset, taking a 20% subset as our holdout set for testing. Using a 5-fold cross-validation strategy, we break the remaining samples into 5 distinct subsets using a 80/20 split for training/validation. We train one model per fold for a maximum of 500 epochs each, and combine the 5 trained models to form an ensemble. While there are many gradient descent optimization algorithms, we use Adam (Kingma and Ba, 2015) as a popular and well-established optimizer, keeping its default configuration and setting our initial learning rate to 0.001. In Figure 4, we show a breakdown of our ensemble pipeline whereby the blue squares highlight the validation subset for each fold. Note, the holdout set is removed before training the ensemble and is only used for testing a complete ensemble.

To estimate the happiness score for a given word, we take a Monte Carlo approach by sampling 100 predictions per model in the ensemble. We use the training setting for the dropout layers in each model, rather than the test time averaging that is commonly used, so that these predictions are heterogeneous. The mean over these predictions becomes the proposed happiness score, while the standard deviation serves as an estimate of model uncertainty (Gal and Ghahramani, 2016). Providing a point estimate along with an uncertainty band allows us to compare and contrast the level of model uncertainty in our ensembles with the uncertainty observed between human annotators.

### 4.2. Comparison With Other Methods and Human Annotators

Although both of our proposed strategies—namely using character-level *n*-grams and word definitions—performed well, the Dictionary model outperforms the Token model. To evaluate our models we train 10 replicates each and then investigate error distributions obtained using the test set. We report the mean absolute error (MAE) as an estimate of overall performance, along with a selection of percentiles to compare tail behavior across models. Each of these statistics are averaged over the 10 replicates. This process provides us with a strong estimate of the generalization performance for our proposed models.

Table 1 summarizes the results of this evaluation process for our proposed models and ensembles. We provide baseline comparisons to models from previous work (Amir et al., 2015; Li et al., 2017), including popular linear models, random forests, and support vector machines trained with three different flavors of word embeddings: Word2Vec (Mikolov et al., 2013a), GloVe (Pennington et al., 2014), and FastText (Bojanowski et al., 2017). These results indicate that our Token model outperforms all prior baselines, our Dictionary model outperforms our Token model, and both of our proposed models benefited from ensemble learning. Though the ensembles outperformed the individual models in both cases, it is interesting to note that they also had longer tails for their error distributions.

**Table 1:** Summary statistics of the testing subset comparing our models to the annotated ratings reported in labMT.

**Mean absolute error (MAE)**		**Percentiles**				
**Model**	**Average**	**25** ***^th^***	**50** ***^th^***	**75** ***^th^***	**85** ***^th^***	**95** ***^th^***
**Linear models**						
ElasticNet + *Word2Vec*	0.81	0.82	0.81	0.82	0.82	0.83
ElasticNet + *GloVe*	0.81	0.82	0.81	0.82	0.82	0.82
ElasticNet + *FastText*	0.81	0.82	0.81	0.82	0.82	0.82
LASSO + *Word2Vec*	0.81	0.81	0.81	0.81	0.82	0.83
LASSO + *GloVe*	0.81	0.81	0.82	0.82	0.82	0.82
LASSO + *FastText*	0.81	0.80	0.81	0.81	0.81	0.82
Ridge + *Word2Vec*	0.73	0.73	0.73	0.74	0.74	0.75
Ridge + *GloVe*	0.75	0.74	0.75	0.75	0.77	0.79
Ridge + *FastText*	0.73	0.73	0.73	0.74	0.74	0.74
**Random forest (RF) models**						
RF + *Word2Vec*	0.69	0.69	0.70	0.70	0.71	0.78
RF + *GloVe*	0.70	0.70	0.70	0.71	0.71	0.71
RF + *FastText*	0.68	0.67	0.68	0.68	0.68	0.69
**Support vector regressor (SVR) models**						
SVR + *Word2Vec*	0.65	0.65	0.65	0.66	0.66	0.67
SVR + *GloVe*	0.67	0.68	0.67	0.66	0.68	0.69
SVR + *FastText*	0.64	0.64	0.64	0.65	0.66	0.66
**Proposed models**						
Token model (single)	0.62	0.60	0.61	0.64	0.65	0.66
Token model (ensemble)	0.57	0.29	0.44	0.66	0.72	0.77
Dictionary model (single)	0.50	0.49	0.50	0.51	**0.51**	**0.52**
Dictionary model (ensemble)	**0.45**	**0.15**	**0.31**	**0.40**	0.52	0.59
Human ratings (standard deviation σ)	1.38	1.18	1.36	1.56	1.69	1.90
Human ratings (variance σ^2^)	1.99	1.39	1.85	2.43	2.86	3.61

*Each word in the labMT lexicon is scored by 50 distinct individuals and the final happiness score is the unweighted mean of their scores (Dodds et al., 2015). We report the standard deviation and variance of the ratings as a baseline to assess the human's confidence in the reported scores. Comparing our predictions with the annotations crowdsourced via AMT, our mean absolute errors are on par with the variance observe in the human annotated labMT scores. In addition to our proposed models, we also evaluate three groups of baseline models based on linear regression, random forests, and support vector machines. We trained and evaluated each baseline model over 10 trials using one of three pre-trained embeddings as the primary input: word2vec-google-news-300 (Mikolov et al., 2013a), glove-wiki-gigaword-300 (Pennington et al., 2014), fasttext-wiki-news-subwords-300 (Bojanowski et al., 2017). Our baseline models are similar to those seen in Amir et al. (2015) and Li et al. (2017). Best models are highlighted in bold*.

**Figure 5:**
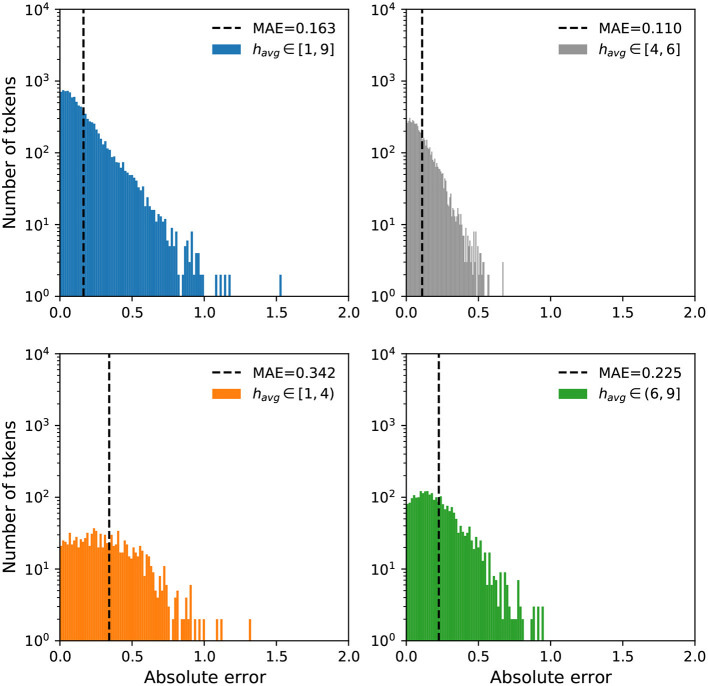
Error distributions for the Token model. We display mean absolute errors for predictions using the Token model on all words in labMT. We arrange the happiness scores into three groups: negative (*h*_*avg*_ ∈ [1,4), orange), neutral (*h*_*avg*_ ∈ [4,6], gray), and positive (*h*_*avg*_ ∈ (6,9], green). Most words have an MAE less than 1 with the exception of a few outliers. We see a relatively higher MAE for negative and positive terms compared to neutral expressions.

**Figure 6:**
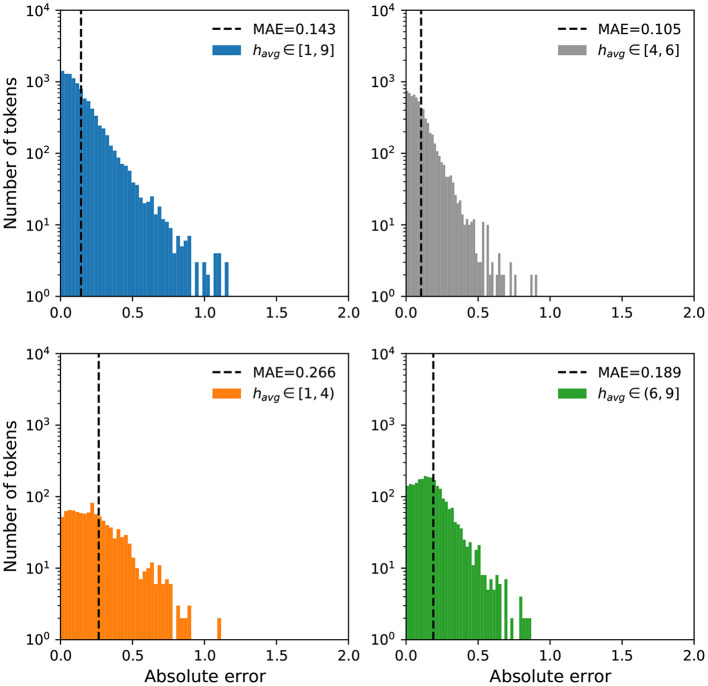
Error distributions for the Dictionary model. We display mean absolute errors for predictions using the Dictionary model on all words in labMT. Again, we categorize the happiness scores into three groups: negative (*h*_*avg*_ ∈ [1,4), orange), neutral (*h*_*avg*_ ∈ [4,6], gray), and positive (*h*_*avg*_ ∈ (6,9], green). Similar to the Token model, most words have an MAE less than 1 with the exception of a few outliers. While the Dictionary model outperforms the Token model, we still observe a higher MAE for negative and positive terms compared to neutral expressions.

We further examine the error distributions to investigate if the models have a bias toward high or low happiness scores. In Figures 5, 6, we display a breakdown of our MAE distributions for the Token and Dictionary models, respectively. For ease of interpretation and visualization, we categorize the happiness scores into three groups: negative (*h*_*avg*_ ∈ [1,4)), neutral (*h*_*avg*_ ∈ [4,6]), and positive (*h*_*avg*_ ∈ (6,9]). While the distributions show our models operate well on all words, particularly neutral expressions, we note a relatively higher MAE for negative words, whereby our predictions to these terms are more positive than the annotations.

We also compare our predictions to the ground-truth ratings, examining the degree to which the models either overshoot or undershoot the happiness scores crowdsourced via AMT. Words in the labMT lexicon were scored by taking the average happiness score of distinct evaluations from 50 different individuals (see Table S2, Dodds et al., 2015). Since the variance of human ratings and our model MAEs are on the same scale, we can use the observed average variance of the ratings (1.17) as a baseline to assess rater confidence in the reported scores. Comparing our models to that baseline, we note that all models offer consistent predictions with similar expectations to a random and reliable reviewer from AMT. See Table 1 for further statistical details.

**Figure 7:**
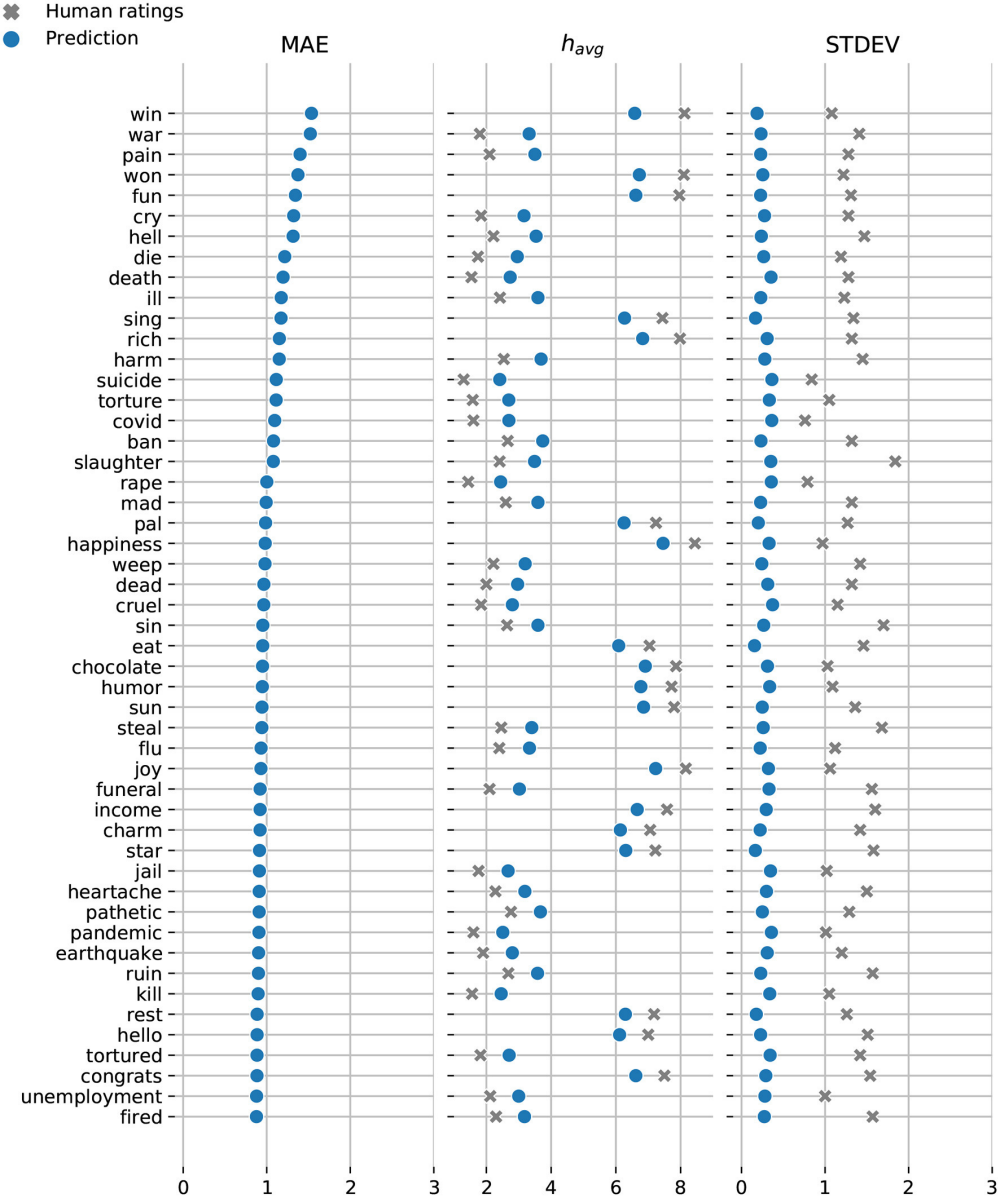
Token model: Top-50 words with the highest mean absolute error. Model predictions are shown in blue and the crowdsourced annotations are displayed in gray. While still maintaining relatively low MAE, most of our predictions are conservative—marginally underestimating words with extremely high happiness scores, and overestimating words with low happiness scores.

**Figure 8:**
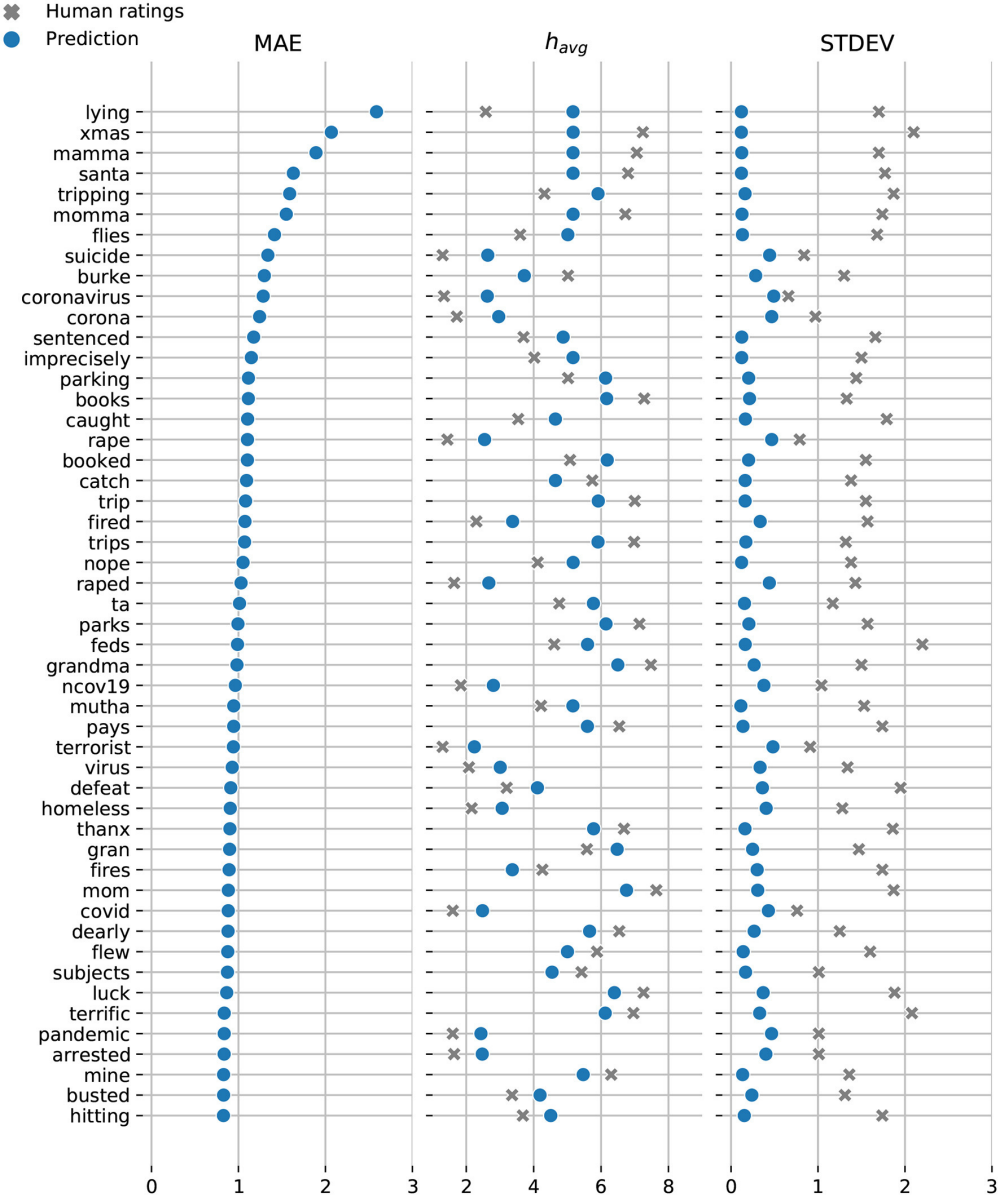
Dictionary model: Top-50 words with the highest mean absolute error. Model predictions are shown in blue and the crowdsourced annotations are displayed in gray. Note, the vast majority of words with relatively high MAE also have high standard deviations of AMT ratings. Words that have multiple definitions will have a neutral score (e.g., lying). A neutral happiness score is also often predicted for words because we are unable to obtain good definitions for them to use as input. Although we have definitions for most words in our dataset, we still have a little over 1,500 words with missing definitions. Most of these words are names (e.g., “‘Burke,”) and slang (e.g., “xmas,” and “ta.”)

In Figures 7, 8, we display the top-50 words with the highest mean absolute error for the Token and Dictionary models, respectively. While the models always predict the right emotional attitude outlining each word based on its lexical polarity, they bias toward neutral by undershooting scores for happy words, and overshooting scores for sad expressions.

One possible explanation of this systematic behavior is the lack of words with extreme happiness scores in the labMT lexicon. It is possible to train models with a smaller but balanced subset of the dataset to overcome that challenge. Doing so, however, would reduce the size of training/validation samples substantially. Still, our margin of error is relatively low compared to human ratings. Future investigations may test and improve the models by examining larger sentiment lexicons.

Another key factor that plays a big role in our prediction error is obtaining good word definitions, or the lack thereof, to use as input for our Dictionary model. Surprisingly, outsourcing definitions from online dictionaries for a large set of words is rather challenging, especially if you opt-out of reliable but paid services. In our work, we choose not to use an urban dictionary or any services with paid APIs. We use a free online dictionary API that is available at https://dictionaryapi.dev.

While we do have definitions for most words in our dataset, a total of 1518 words have missing definitions. Most of these words are names, abbreviations, and slang terms (e.g., “xams,” “foto,” “nvm,” and “lmao”). Words with multiple definitions can also cancel each other's score (e.g., “lying”).

Notably, the vast majority of words with high MAE also have high AMT standard deviations. To further investigate prediction accuracy, we examine the overlap between the predictions and human ratings. In particular, we compute the intersection over union (IOU) between the predicted happiness score $h_{avg}^{\prime }\pm \sigma^{\prime }$, and the corresponding value from the annotated ratings *h*_*avg*_±σ.

The Token model underestimates the happiness score for “win”—the only word with a prediction that falls outside the range of human annotated happiness scores. The remaining predicted happiness scores fall well within the range of scores crowdsourced via AMT. Similarly, the Dictionary model slightly underestimates the happiness scores for “mamma” while overestimating the scores for “lying,” and “coronavirus.”

## 5. Discussion

As the growing demand for sentiment-aware intelligent systems increases, we will continue to see improvements to both lexicon-based models and contextual language models. While contextualized models are suitable for a wide set of applications, lexicon-based models are used by computational linguists, journalists, and data scientists who are interested in studying how individual words contribute to sentiment trends.

Sentiment lexicons, however, have to be updated periodically to support new words and expressions that were not considered when the dictionaries were assembled. In this paper, we proposed two models for predicting sentiment scores to augment semantic dictionaries using word embeddings and pre-trained large language models. Our first model establishes a baseline using a neural network initialized with pre-trained word embeddings, while our second model features a deep Transformer-based network that brings into play word definitions to estimate their lexical polarity. Our results and evaluation of both models demonstrate human-level performance on a state-of-the-art human annotated list of words.

Although both models can predict scores for novel words, we acknowledge a few shortcomings. Our Token model relies on subword information to estimate a happiness score for any given word. For example, using subwords for “coronavirus” yields a good estimate given that it contains “virus.” By contrast, parsing character-level *n*-grams for other words (e.g., “covid”) may not reveal any further information. We can overcome that hurdle by using the word definition as input to our Dictionary model to gauge its happiness score. Words, however, often have different meanings based on context. Finding good definitions may be challenging, especially for slang, informal expressions, and abbreviations. We recommend using the Dictionary model whenever it is possible to outsource a good definition of the word.

A natural next step would be to develop similar models for other languages, for example by building a model for each language, or a multilingual model. Fortunately, FastText (Bojanowski et al., 2017) provides pre-trained word embeddings for over 100 languages. Therefore, it is easy to upgrade the Token model to support other languages. Updating the Dictionary model is also a straightforward task by simply adopting a multilingual Transformer-based model pre-trained with several languages [e.g., Multilingual BERT (Devlin et al., 2019)]. We caution against translating words and using the same English scores because most words do not have a one-to-one mapping into other languages, and are often used to express different meanings by the native speakers of any given language (Dodds et al., 2015).

Another vast space of improvements would be to adopt our proposed strategies to develop prediction models for other semantic dictionaries. Researchers can further fine-tune these models to predict other sentiment scores. For example, the happiness scores in the labMT (Dodds et al., 2015) dataset are closely aligned with the valence scores in the NRC-VAD lexicon (Mohammad, 2018). We envision future work developing similar models to predict other semantic differentials such as arousal and dominance (Mohammad, 2018), EPA (Osgood, 1962), and SocialSent (Hamilton et al., 2016). Our primary goal is to provide an easy and robust method to augment semantic dictionaries to empower researchers to maintain and expand them at a relatively low cost using today's state-of-the-art NLP methods.

## Data Availability Statement

Our source code along with pre-trained models are publicly available on our Gitlab repository (https://gitlab.com/compstorylab/sentiment-analysis).

## Author Contributions

TA designed and developed the methods. TA and CV verified and analyzed data. TA, CV, MF, MA, CD, and PD edited the manuscript. CD and PD supervised the project. All authors provided critical feedback and helped shape the research, analysis and manuscript.

## Funding

We are grateful for the computing resources provided by the Vermont Advanced Computing Core and financial support from Google and the Massachusetts Mutual Life Insurance Company. Computations were performed on the Vermont Advanced Computing Core supported in part by NSF award No. OAC-1827314.

## Conflict of Interest

The authors declare that the research was conducted in the absence of any commercial or financial relationships that could be construed as a potential conflict of interest.

## Publisher's Note

All claims expressed in this article are solely those of the authors and do not necessarily represent those of their affiliated organizations, or those of the publisher, the editors and the reviewers. Any product that may be evaluated in this article, or claim that may be made by its manufacturer, is not guaranteed or endorsed by the publisher.

## References

[B1] Abadi M. Barham P. Chen J. Chen Z. Davis A. Dean J.. (2016). Tensorflow: a system for large-scale machine learning, in Proceedings of the 12th USENIX Conference on Operating Systems Design and Implementation OSDI'16 (Berkeley, CA: USENIX Association), 265–283.

[B2] Agarwal A. Xie B. Vovsha I. Rambow O. Passonneau R. (2011). Sentiment analysis of Twitter data, in Proceedings of the Workshop on Language in Social Media (LSM 2011) (Portland: Association for Computational Linguistics), 30–38.

[B3] Aithal M. Tan C. (2021). On positivity bias in negative reviews, in Proceedings of the Joint Conference of the 59th Annual Meeting of the Association for Computational Linguistics and the 11th International Joint Conference on Natural Language Processing (ACL-IJCNLP 2021) (Association for Computational Linguistics).

[B4] Alshaabi T. Adams J. L. Arnold M. V. Minot J. R. Dewhurst D. R. Reagan A. J.. (2021a). Storywrangler: a massive exploratorium for sociolinguistic, cultural, socioeconomic, and political timelines using Twitter. Sci. Adv. 7:eabe6534. 10.1126/sciadv.abe6534 34272243 PMC8284897

[B5] Alshaabi T. Arnold M. V. Minot J. R. Adams J. L. Dewhurst D. R. Reagan A. J.. (2021b). How the world's collective attention is being paid to a pandemic: COVID-19 related n-gram time series for 24 languages on Twitter. PLoS One 16:e0244476. 10.1371/journal.pone.0244476 33406101 PMC7787459

[B6] Alshari E. M. Azman A. Doraisamy S. Mustapha N. Alkeshr M. (2018). Effective method for sentiment lexical dictionary enrichment based on Word2Vec for sentiment analysis, in 2018 Fourth International Conference on Information Retrieval and Knowledge Management (CAMP) (Kota Kinabalu), 1–5.

[B7] Amir S. F. Astudillo R. Ling W. Martins B. Silva M. J. Trancoso I. (2015). INESC-ID: a regression model for large scale Twitter sentiment lexicon induction, in Proceedings of the 9th International Workshop on Semantic Evaluation (SemEval 2015) (Denver, CO: Association for Computational Linguistics), 613–618.

[B8] Augustyniak Ł. Szymański P. Kajdanowicz T. Tuligłowicz W. (2016). Comprehensive study on lexicon-based ensemble classification sentiment analysis. Entropy 18:4. 10.3390/e18010004

[B9] Baccianella S. Esuli A. Sebastiani F. (2010). SentiWordNet 3.0: An enhanced lexical resource for sentiment analysis and opinion mining, in Proceedings of the Seventh International Conference on Language Resources and Evaluation (LREC'10) (Valletta: European Language Resources Association (ELRA)).

[B10] Bajpai R. Ho D. Cambria E. (2016). Developing a concept-level knowledge base for sentiment analysis in singlish, in International Conference on Intelligent Text Processing and Computational Linguistics (Konya: Springer), 347–361.

[B11] Bakshi R. K. Kaur N. Kaur R. Kaur G. (2016). Opinion mining and sentiment analysis, in 2016 3rd international Conference on Computing for Sustainable Global Development (INDIACom) (New Delhi: IEEE), 452–455.

[B12] Bathina K. C. Ten Thij M. Lorenzo-Luaces L. Rutter L. A. Bollen J. (2021). Individuals with depression express more distorted thinking on social media. Nat. Human Behav. 5, 458–466. 10.1038/s41562-021-01050-7 33574604

[B13] Beigi G. Hu X. Maciejewski R. Liu H. (2016). An overview of sentiment analysis in social media and its applications in disaster relief, in Sentiment Analysis and Ontology Engineering (Cham: Springer), 313–340.

[B14] Bengio Y. Ducharme R. Vincent P. Janvin C. (2003). A neural probabilistic language model. J. Mach. Learn. Res. 3, 1137–1155. 10.5555/944919.94496626215079

[B15] Bhatt A. Patel A. Chheda H. Gawande K. (2015). Amazon review classification and sentiment analysis. Int. J. Comput. Sci. Inf. Technol. 6, 5107–5110.

[B16] Bojanowski P. Grave E. Joulin A. Mikolov T. (2017). Enriching word vectors with subword information. Trans. Assoc. Comput. Linguist. 5, 135–146. 10.1162/tacl_a_00051

[B17] Bradley M. M. Lang P. J. (1999). Affective Norms for English Words (ANEW): Instruction Manual and Affective Ratings. Technical Report, Technical Report C-1, Gainesville, FL: Center for Research in Psychophysiology.

[B18] Cabral L. Hortacsu A. (2010). The dynamics of seller reputation: evidence from eBay. J. Ind. Econ. 58, 54–78. 10.1111/j.1467-6451.2010.00405.x

[B19] Cambria E. Li Y. Xing F. Z. Poria S. Kwok K. (2020). Senticnet 6: ensemble application of symbolic and subsymbolic ai for sentiment analysis, in Proceedings of the 29th ACM international conference on information & knowledge management 105–114.

[B20] Chen H. Yang C. Zhang X. Liu Z. Sun M. Jin J. (2021). From Symbols to Embeddings: a Tale of Two Representations in Computational Social Science. Available online at: https://arxiv.org/abs/2106.14198

[B21] Chen Q. Zhu X. Ling Z.-H. Wei S. Jiang H. Inkpen D. (2017). Enhanced LSTM for natural language inference, in Proceedings of the 55th Annual Meeting of the Association for Computational Linguistics (Volume 1: Long Papers) (Vancouver, BC: Association for Computational Linguistics), 1657–1668.

[B22] Colhon M. Vlăduţescu Ş. Negrea X. (2017). How objective a neutral word is? a neutrosophic approach for the objectivity degrees of neutral words. Symmetry 9:280. 10.3390/sym9110280

[B23] Conway M. O'Connor D. (2016). Social media, big data, and mental health: current advances and ethical implications. Curr. Opin. Psychol. 9, 77–82. 10.1016/j.copsyc.2016.01.004 27042689 PMC4815031

[B24] Coppersmith G. Dredze M. Harman C. (2014). Quantifying mental health signals in Twitter, in Proceedings of the Workshop on Computational Linguistics and Clinical Psychology: From Linguistic Signal to Clinical Reality (Baltimore, MD), 51–60.

[B25] Crawford K. (2019). Halt the use of facial-recognition technology until it is regulated. Nature 572, 565–566. 10.1038/d41586-019-02514-7 31455918

[B26] Crawford K. Paglen T. (2021). Excavating ai: the politics of images in machine learning training sets. AI & SOCIETY 9, 1–12. 10.1007/s00146-021-01162-8

[B27] Dai Z. Yang Z. Yang Y. Carbonell J. Le Q. Salakhutdinov R. (2019). Transformer-XL: attentive language models beyond a fixed-length context, in Proceedings of the 57th Annual Meeting of the Association for Computational Linguistics (Florence: Association for Computational Linguistics), 2978–2988.

[B28] Darwich M. Mohd Noah S. A. Omar N. Osman N. A. (2019). Corpus-based techniques for sentiment lexicon generation: a review. J. Digit. Inf. Manag. 17, 296. 10.6025/jdim/2019/17/5/296-305

[B29] Devlin J. Chang M.-W. Lee K. Toutanova K. (2019). BERT: pre-training of deep bidirectional transformers for language understanding, in Proceedings of the 2019 Conference of the North American Chapter of the Association for Computational Linguistics: Human Language Technologies, Volume 1 (Long and Short Papers) (Minneapolis: Association for Computational Linguistics), 4171–4186.

[B30] Dodds P. S. Clark E. M. Desu S. Frank M. R. Reagan A. J. Williams J. R.. (2015). Human language reveals a universal positivity bias. Proc. Natl. Acad. Sci. U.S.A. 112, 2389–2394. 10.1073/pnas.1411678112 25675475 PMC4345622

[B31] Dodds P. S. Danforth C. M. (2010). Measuring the happiness of large-scale written expression: songs, blogs, and presidents. J. Happiness Stud. 11, 441–456. 10.1007/s10902-009-9150-9

[B32] Dodds P. S. Harris K. D. Kloumann I. M. Bliss C. A. Danforth C. M. (2011). Temporal patterns of happiness and information in a global social network: hedonometrics and Twitter. PLoS One 6:e26752. 10.1371/journal.pone.0026752 22163266 PMC3233600

[B33] Dosovitskiy A. Beyer L. Kolesnikov A. Weissenborn D. Zhai X. Unterthiner T.. (2021). An image is worth 16x16 words: transformers for image recognition at scale, in Proceedings of the International Conference on Learning Representations ICRL'21.

[B34] Dowlagar S. Mamidi R. (2021). Graph convolutional networks with multi-headed attention for code-mixed sentiment analysis, in Proceedings of the First Workshop on Speech and Language Technologies for Dravidian Languages (Kyiv), 65–72.

[B35] Feldman R. (2013). Techniques and applications for sentiment analysis. Commun. ACM 56, 82–89. 10.1145/2436256.2436274

[B36] Fellbaum C. editor (1998). Language, Speech, and Communication. A Bradford Book, in WordNet: An Electronic Lexical Database (Cambridge, MA: A Bradford Book).

[B37] Gal Y. Ghahramani Z. (2016). Dropout as a bayesian approximation: representing model uncertainty in deep learning, in Balcan M. F. Weinberger K. Q. editors. Proceedings of The 33rd International Conference on Machine Learning, Proceedings of Machine Learning Research Vol. 48. (New York, NY: PMLR), 1050–1059.

[B38] Gallagher R. J. Frank M. R. Mitchell L. Schwartz A. J. Reagan A. J. Danforth C. M.. (2021). Generalized word shift graphs: a method for visualizing and explaining pairwise comparisons between texts. EPJ Data Sci. 10:4. 10.1140/epjds/s13688-021-00260-3

[B39] Gohil S. Vuik S. Darzi A. (2018). Sentiment analysis of health care tweets: review of the methods used. JMIR Public Health Surveillance 4:e43. 10.2196/publichealth.5789 29685871 PMC5938573

[B40] Hamilton W. L. Clark K. Leskovec J. Jurafsky D. (2016). Inducing domain-specific sentiment lexicons from unlabeled corpora, in Proceedings of the 2016 Conference on Empirical Methods in Natural Language Processing (Austin, TX: Association for Computational Linguistics), 595–605. 10.18653/v1/D16-1057 PMC5483533 28660257

[B41] Hansen L. K. Salamon P. (1990). Neural network ensembles. IEEE Trans. Pattern Anal. Mach. Intell. 12, 993–1001. 10.1109/34.5887127295638

[B42] Haque T. U. Saber N. N. Shah F. M. (2018). Sentiment analysis on large scale Amazon product reviews, in 2018 IEEE international conference on innovative research and development (ICIRD) (Bangkok: IEEE), 1–6.

[B43] Harris Z. S. (1954). Distributional structure. Word 10, 146–162. 10.1080/00437956.1954.11659520

[B44] Hinton G. Vinyals O. Dean J. (2015). Distilling the knowledge in a neural network, in NIPS Deep Learning and Representation Learning Workshop. Montreal, QC.

[B45] Hochreiter S. Schmidhuber J. (1997). Long short-term memory. Neural Comput. 9, 1735–1780. 9377276 10.1162/neco.1997.9.8.1735

[B46] Hoerl A. E. Kennard R. W. (1970). Ridge regression: biased estimation for nonorthogonal problems. Technometrics 12, 55–67. 10.2307/1271436

[B47] Hollis G. Westbury C. (2016). The principals of meaning: extracting semantic dimensions from co-occurrence models of semantics. Psychon. Bull. Rev. 23, 1744–1756. 10.3758/S13423-016-1053-2 27138012

[B48] Hollis G. Westbury C. Lefsrud L. (2017). Extrapolating human judgments from skip-gram vector representations of word meaning. Quart. J. Exp. Psychol. 70, 1603–1619. 10.1080/17470218.2016.1195417 27251936

[B49] Hovy D. Spruit S. L. (2016). The social impact of natural language processing, in Proceedings of the 54th Annual Meeting of the Association for Computational Linguistics (Volume 2: Short Papers) Berlin, 591–598.

[B50] Joulin A. Grave E. Bojanowski P. Mikolov T. (2017). Bag of tricks for efficient text classification, in Proceedings of the 15th Conference of the European Chapter of the Association for Computational Linguistics: Volume 2, Short Papers (Valencia: Association for Computational Linguistics), 427–431.

[B51] Kingma D. P. Ba J. (2015). Adam: a method for stochastic optimization, in Bengio Y. LeCun Y. editors, 3rd International Conference on Learning Representations, ICLR Conference Track Proceedings (San Diego, CA: ICLR).

[B52] Kiritchenko S. Zhu X. Mohammad S. M. (2014). Sentiment analysis of short informal texts. J. Artif. Intell. Res. 50, 723–762. 10.1613/jair.4272

[B53] Kissler J. Herbert C. (2013). Emotion, etmnooi, or emitoon?—faster lexical access to emotional than to neutral words during reading. Biol. Psychol. 92, 464–479. 10.1016/j.biopsycho.2012.09.004 23059636

[B54] Koppel M. Schler J. (2006). The importance of neutral examples for learning sentiment. Comput. Intell. 22, 100–109. 10.1111/j.1467-8640.2006.00276.x23255960

[B55] Korkontzelos I. Nikfarjam A. Shardlow M. Sarker A. Ananiadou S. Gonzalez G. H. (2016). Analysis of the effect of sentiment analysis on extracting adverse drug reactions from tweets and forum posts. J. Biomed. Informat. 62, 148–158. 10.1016/j.jbi.2016.06.007 27363901 PMC4981644

[B56] Krogh A. Vedelsby J. (1994). Neural network ensembles, cross validation and active learning, in Proceedings of the 7th International Conference on Neural Information Processing Systems NIPS'94 (Cambridge, MA: MIT Press), 231–238.

[B57] Kudo T. Richardson J. (2018). SentencePiece: a simple and language independent subword tokenizer and detokenizer for neural text processing, in Proceedings of the 2018 Conference on Empirical Methods in Natural Language Processing: System Demonstrations (Brussels: Association for Computational Linguistics), 66–71.

[B58] Kumar A. Lee C. M. (2006). Retail investor sentiment and return comovements. J. Finance 61, 2451–2486. 10.1111/j.1540-6261.2006.01063.x

[B59] Lan Z. Chen M. Goodman S. Gimpel K. Sharma P. Soricut R. (2020). ALBERT: a lite BERT for self-supervised learning of language representations, in Proceedings of the International Conference on Learning Representations.

[B60] Laver M. Benoit K. Garry J. (2003). Extracting policy positions from political texts using words as data. Amer. Polit. Sci. Rev. 97, 311–331. 10.1017/S000305540300069830886898

[B61] Lee K. He L. Lewis M. Zettlemoyer L. (2017). End-to-end neural coreference resolution, in Proceedings of the 2017 Conference on Empirical Methods in Natural Language Processing (Copenhagen: Association for Computational Linguistics), 188–197.

[B62] Li M. Lu Q. Long Y. Gui L. (2017). Inferring affective meanings of words from word embedding. IEEE Trans. Affect. Comput. 8, 443–456. 10.1109/TAFFC.2017.272301227295638

[B63] Liao W. Zeng B. Liu J. Wei P. Cheng X. Zhang W. (2021). Multi-level graph neural network for text sentiment analysis. Comput. Elect. Eng. 92:107096. 10.1016/j.compeleceng.2021.107096

[B64] Ljubešić N. Fišer D. Peti-Stantić A. (2018). Predicting concreteness and imageability of words within and across languages via word embeddings, in Proceedings of The Third Workshop on Representation Learning for NLP (Melbourne, VIC: Association for Computational Linguistics), 217–222.

[B65] Maas A. L. Daly R. E. Pham P. T. Huang D. Ng A. Y. Potts C. (2011). Learning word vectors for sentiment analysis, in Proceedings of the 49th Annual Meeting of the Association for Computational Linguistics: Human Language Technologies (Portland, OR: Association for Computational Linguistics), 142–150.

[B66] Mayzner M. S. Tresselt M. E. (1965). Tables of single-letter and digram frequency counts for various word-length and letter-position combinations. Psychonomic Monograph Supplements 1, 13–32.

[B67] Medhat W. Hassan A. Korashy H. (2014). Sentiment analysis algorithms and applications: a survey. Ain Shams Eng. J. 5, 1093–1113. 10.1016/j.asej.2014.04.011

[B68] Mikolov T. Chen K. Corrado G. Dean J. (2013a). Efficient estimation of word representations in vector space. In Bengio Y. LeCun Y. editors, 1st International Conference on Learning Representations, ICLR 2013, Scottsdale, Arizona, USA, May 2-4, 2013, Workshop Track Proceedings.

[B69] Mikolov T. Sutskever I. Chen K. Corrado G. Dean J. (2013b). Distributed representations of words and phrases and their compositionality, in Proceedings of the 26th International Conference on Neural Information Processing Systems - Volume 2, NIPS'13 (Red Hook, NY: Curran Associates Inc.), 3111–3119.

[B70] Miller G. A. Newman E. B. Friedman E. A. (1958). Length-frequency statistics for written English. Inf. Control 1, 370–389. 10.1016/S0019-9958(58)90229-8

[B71] Mohammad S. (2018). Obtaining reliable human ratings of valence, arousal, and dominance for 20,000 English words, in Proceedings of the 56th Annual Meeting of the Association for Computational Linguistics (Volume 1: Long Papers) (Melbourne, VIC: Association for Computational Linguistics), 174–184.

[B72] Nasukawa T. Yi J. (2003). Sentiment analysis: capturing favorability using natural language processing, in Proceedings of the 2nd International Conference on Knowledge Capture (New York, NY), 70–77.

[B73] Osgood C. E. (1962). Studies on the generality of affective meaning systems. Amer. Psychol. 17:10. 10.1037/h0045146 14316974

[B74] Pak A. Paroubek P. (2010). Twitter as a corpus for sentiment analysis and opinion mining, in Proceedings of the Seventh International Conference on Language Resources and Evaluation (LREC'10) Valletta: European Language Resources Association (ELRA).

[B75] Pang B. Lee L. (2008). Opinion mining and sentiment analysis. Found. Trends Inf. Retrieval 2, 1–135. 10.1561/1500000011

[B76] Pang B. Lee L. Vaithyanathan S. (2002). Thumbs up? sentiment classification using machine learning techniques, in Proceedings of the ACL-02 Conference on Empirical Methods in Natural Language Processing - Volume 10 EMNLP '02 (Philadelphia, PA: Association for Computational Linguistics), 79–86

[B77] Pennington J. Socher R. Manning C. (2014). GloVe: Global vectors for word representation, in Proceedings of the 2014 Conference on Empirical Methods in Natural Language Processing (EMNLP) (Doha: Association for Computational Linguistics), 1532–1543.

[B78] Peters M. Ammar W. Bhagavatula C. Power R. (2017). Semi-supervised sequence tagging with bidirectional language models, in Proceedings of the 55th Annual Meeting of the Association for Computational Linguistics (Volume 1: Long Papers) (Vancouver, BC: Association for Computational Linguistics), 1756–176.

[B79] Peters M. Neumann M. Iyyer M. Gardner M. Clark C. Lee K. Zettlemoyer L. (2018). Deep contextualized word representations, in Proceedings of the 2018 Conference of the North American Chapter of the Association for Computational Linguistics: Human Language Technologies, Volume 1 (Long Papers) (New Orleans: Association for Computational Linguistics), 2227–2237.

[B80] Qiu G. Liu B. Bu J. Chen C. (2009). Expanding domain sentiment lexicon through double propagation, in Proceedings of the 21st International Joint Conference on Artificial Intelligence IJCAI'09 (Pasadena, CA), 1199–1204.

[B81] Radford A. Narasimhan K. Salimans T. Sutskever I. (2018). Improving language understanding by generative pre-training. Available online at: https://www.cs.ubc.ca/~amuham01/LING530/papers/radford2018improving.pdf

[B82] Reagan A. J. Danforth C. M. Tivnan B. Williams J. R. Dodds P. S. (2017). Sentiment analysis methods for understanding large-scale texts: a case for using continuum-scored words and word shift graphs. EPJ Data Sci. 6, 1–21. 10.1140/epjds/s13688-017-0121-932355601

[B83] Ribeiro F. N. Araújo M. Gonçalves P. Gonçalves M. A. Benevenuto F. (2016). SentiBench - a benchmark comparison of state-of-the-practice sentiment analysis methods. EPJ Data Sci. 5, 1–29. 10.1140/epjds/s13688-016-0085-1

[B84] Riloff E. (1996). An empirical study of automated dictionary construction for information extraction in three domains. Artif. Intell. 85, 101–134. 10.1016/0004-3702(95)00123-9

[B85] Rumelhart D. E. Hinton G. E. Williams R. J. (1986). Learning Internal Representations by Error Propagation. (Cambridge, MA: MIT Press). 318–362.

[B86] San Vicente I. Agerri R. Rigau G. (2014). Simple, robust and (almost) unsupervised generation of polarity lexicons for multiple languages, in Proceedings of the 14th Conference of the European Chapter of the Association for Computational Linguistics (Gothenburg: Association for Computational Linguistics), 88–97.

[B87] Sanh V. Debut L. Chaumond J. Wolf T. (2019). DistilBERT, a distilled version of BERT: smaller, faster, cheaper and lighter, in Proceedings of the 7th International Conference on Neural Information Processing Systems, 5th Workshop on Energy Efficient Machine Learning and Cognitive Computing Vancouver, BC: MIT Press.

[B88] Sennrich R. Haddow B. Birch A. (2016). Neural machine translation of rare words with subword units, in Proceedings of the 54th Annual Meeting of the Association for Computational Linguistics (Volume 1: Long Papers) (Berlin: Association for Computational Linguistics), 1715–1725.

[B89] Shmueli B. Fell J. Ray S. Ku L.-W. (2021). Beyond fair pay: Ethical implications of NLP crowdsourcing, in Proceedings of the 2021 Conference of the North American Chapter of the Association for Computational Linguistics: Human Language Technologies (Association for Computational Linguistics), 3758–3769.

[B90] Snyder B. Barzilay R. (2007). Multiple aspect ranking using the good grief algorithm, in Human Language Technologies 2007: The Conference of the North American Chapter of the Association for Computational Linguistics; Proceedings of the Main Conference (Rochester, NY: Association for Computational Linguistics), 300–307

[B91] Socher R. Chen D. Manning C. D. Ng A. (2013a). Reasoning with neural tensor networks for knowledge base completion, in Advances in Neural Information Processing Systems (Lake Tahoe, NV: Curran Associates Inc.), 926–934.

[B92] Socher R. Perelygin A. Wu J. Chuang J. Manning C. D. Ng A.. (2013b). Recursive deep models for semantic compositionality over a sentiment treebank, in Proceedings of the 2013 Conference on Empirical Methods in Natural Language Processing (Seattle, WA: Association for Computational Linguistics), 1631–1642.

[B93] Srivastava N. Hinton G. Krizhevsky A. Sutskever I. Salakhutdinov R. (2014). Dropout: a simple way to prevent neural networks from overfitting. J. Mach. Learn. Res. 15, 1929–1958. 10.5555/2627435.267031326215079

[B94] Stupinski A. M. Alshaabi T. Arnold M. V. Adams J. L. Minot J. R. Price M.. (2021). Quantifying Language Changes Surrounding Mental Health on Twitter. Available online at: https://arxiv.org/abs/2106.01481

[B95] Taboada M. Brooke J. Tofiloski M. Voll K. Stede M. (2011). Lexicon-based methods for sentiment analysis. Comput. Linguist. 37, 267–307. 10.1162/COLI_a_00049

[B96] Tang D. Wei F. Qin B. Zhou M. Liu T. (2014). `Building large-scale Twitter-specific sentiment lexicon: A representation learning approach, in Proceedings of COLING 2014, the 25th International Conference on Computational Linguistics: Technical Papers (Dublin: Dublin City University and Association for Computational Linguistics), 172–182.

[B97] Tang H. Tan S. Cheng X. (2009). A survey on sentiment detection of reviews. Exp. Syst. Appl. 36, 10760–10773. 10.1016/j.eswa.2009.02.063

[B98] Tatman R. (2017). Gender and dialect bias in YouTube's automatic captions, in Proceedings of the First ACL Workshop on Ethics in Natural Language Processing (Valencia: Association for Computational Linguistics), 53–59.

[B99] Terveen L. Hill W. Amento B. McDonald D. Creter J. (1997). PHOAKS: a system for sharing recommendations. Commun. ACM 40, 59–62. 10.1145/245108.245122

[B100] Thavareesan S. Mahesan S. (2020). Sentiment lexicon expansion using word2vec and fastText for sentiment prediction in Tamil texts, in 2020 Moratuwa Engineering Research Conference (MERCon) (Moratuwa), 272–276.

[B101] Thelwall M. Buckley K. Paltoglou G. Cai D. Kappas A. (2010). Sentiment strength detection in short informal text. J. Amer. Soc. Inf. Sci. Technol. 61, 2544–2558. 10.1002/asi.2141625855820

[B102] Thomas M. Pang B. Lee L. (2006). Get out the vote: determining support or opposition from congressional floor-debate transcripts, in Proceedings of the 2006 Conference on Empirical Methods in Natural Language Processing (Sydney, NSW: Association for Computational Linguistics), 327–335.

[B103] Thompson N. C. Greenewald K. Lee K. Manso G. F. (2020). The Computational Limits of Deep Learning. Available online at: https://arxiv.org/abs/2007.05558

[B104] Tumasjan A. Sprenger T. Sandner P. Welpe I. (2010). Predicting elections with Twitter: what 140 characters reveal about political sentiment, in Proceedings of the International AAAI Conference on Web and Social Media, vol. 4, Washington, DC.

[B105] Turney P. D. (2002). Thumbs up or thumbs down? Semantic orientation applied to unsupervised classification of reviews, in Proceedings of the 40th Annual Meeting on Association for Computational Linguistics ACL '02 (Philadelphia, PA: Association for Computational Linguistics), 417–424.

[B106] Turney P. D. Littman M. L. (2003). Measuring praise and criticism: Inference of semantic orientation from association. ACM Trans. Inf. Syst. 21, 315–346. 10.1145/944012.944013

[B107] Vaswani A. Shazeer N. Parmar N. Uszkoreit J. Jones L. Gomez A. N.. (2017). Attention is all you need, in Advances in Neural Information Processing Systems, eds Guyon I. Luxburg U. V. Bengio S. Wallach H. Fergus R. Vishwanathan S. Garnett R. Vol. 30. (Long Beach, CA: Curran Associates, Inc.).

[B108] Wang J. Yu L.-C. Lai K. R. Zhang X. (2016). Community-based weighted graph model for valence-arousal prediction of affective words. IEEE/ACM Trans. Audio Speech Lang. Process. 24, 1957–1968. 10.1109/TASLP.2016.259428727295638

[B109] Wilson T. Wiebe J. Hoffmann P. (2005). Recognizing contextual polarity in phrase-level sentiment analysis, in Proceedings of the Conference on Human Language Technology and Empirical Methods in Natural Language Processing HLT '05 (Vancouver, BC: Association for Computational Linguistics), 347–354.

[B110] Wolf T. Debut L. Sanh V. Chaumond J. Delangue C. Moi A.. (2020). Transformers: state-of-the-art natural language processing, in Proceedings of the 2020 Conference on Empirical Methods in Natural Language Processing: System Demonstrations (Association for Computational Linguistics), 38–45.

[B111] Wu Y. Schuster M. Chen Z. Le Q. V. Norouzi M. Macherey W.. (2016). Google's Neural Machine Translation System: Bridging the Gap Between Human and Machine Translation. Available online at: https://arxiv.org/abs/1609.08144

[B112] Yadollahi A. Shahraki A. G. Zaiane O. R. (2017). Current state of text sentiment analysis from opinion to emotion mining. ACM Comput. Surveys 50, 1–33. 10.1145/3057270

[B113] Yang S. Xing L. Li Y. Chang Z. (2021). Implicit sentiment analysis based on graph attention neural network. Eng. Rep. 2012:e12452. 10.1002/eng2.1245225855820

[B114] Yang Z. Dai Z. Yang Y. Carbonell J. Salakhutdinov R. R. Le Q. V. (2019). Xlnet: generalized autoregressive pretraining for language understanding, in Advances in Neural Information Processing Systems, Vol. 32. (Vancouver, BC: Curran Associates, Inc.).32723719

[B115] Yu Y. Duan W. Cao Q. (2013). The impact of social and conventional media on firm equity value: a sentiment analysis approach. Decis. Support Syst. 55, 919–926. 10.1016/j.dss.2012.12.028

[B116] Yuan M. Lin Y. (2006). Model selection and estimation in regression with grouped variables. J. Roy. Stat. Soc. B (Stati. Methodol.) 68, 49–67. 10.1111/J.1467-9868.2005.00532.X28192605

[B117] Zou H. Hastie T. (2005). Regularization and variable selection via the elastic net. J. Roy. Stat. Soc. B (Stati. Methodol.) 67, 301–320. 10.1111/j.1467-9868.2005.00503.x

